# Perinuclear compartment controls calcineurin/MEF2 signaling for axonal outgrowth of hippocampal neurons

**DOI:** 10.3389/fnmol.2024.1494160

**Published:** 2024-11-25

**Authors:** Joanna Mackiewicz, Malwina Lisek, Julia Tomczak, Agata Sakowicz, Feng Guo, Tomasz Boczek

**Affiliations:** ^1^Department of Molecular Neurochemistry, Medical University of Lodz, Lodz, Poland; ^2^Department of Medical Biotechnology, Medical University of Lodz, Lodz, Poland; ^3^Department of Pharmaceutical Toxicology, China Medical University, Shenyang, China; ^4^Department of Pharmacy, The Fourth Affiliated Hospital of China Medical University, Shenyang, China

**Keywords:** mAKAP signalosome, hippocampal neurons, calcineurin, MEF2, axonal outgrowth, neuronal development, perinuclear space

## Abstract

Central to the process of axon elongation is the concept of compartmentalized signaling, which involves the A-kinase anchoring protein (AKAP)-dependent organization of signaling pathways within distinct subcellular domains. This spatial organization is also critical for translating electrical activity into biochemical events. Despite intensive research, the detailed mechanisms by which the spatial separation of signaling pathways governs axonal outgrowth and pathfinding remain unresolved. In this study, we demonstrate that mAKAPα (AKAP6), located in the perinuclear space of primary hippocampal neurons, scaffolds calcineurin, NFAT, and MEF2 transcription factors for activity-dependent axon elongation. By employing anchoring disruptors, we show that the mAKAPα/calcineurin/MEF2 signaling pathway, but not NFAT, drives the process of axonal outgrowth. Furthermore, mAKAPα-controlled axonal elongation is linked to the changes in the expression of genes involved in Ca^2+^/cAMP signaling. These findings reveal a novel regulatory mechanism of axon growth that could be targeted therapeutically for neuroprotection and regeneration.

## 1 Introduction

A unique and fundamental characteristic of the nervous system during development is the ability of neurons to extend long axonal protrusions in response to specific environmental cues. Due to the remarkable conservation of the growth machinery between development and adulthood (Bradke, 2022), reactivating the processes that promote axonal growth in mature neurons could enable the regeneration of injured axons. Recent attention has focused on compartmentalized mechanisms of neuronal signaling that promote the survival and regeneration of neurons, aiming to dissect defective transduction pathways that could be therapeutically targeted. However, in light of the compartmentalized theory of neuronal signaling, little is still known about the processes that control axonal outgrowth and pathfinding. The role of electrical activity in regulating axonal elongation during development is also a subject of debate.

According to this theory, neurons possess highly specialized centers for integrating multiple, sometimes contradictory, signals, allowing for a coordinated response to a given stimulus. The fidelity and specificity of the signaling are ensured by the spatial tethering of signaling components by a nodal scaffold protein called A-kinase Anchoring Protein (AKAP) (Wang et al., 2015; Wild and Dell'Acqua, 2018; Boczek et al., 2021; Dodge-Kafka et al., 2005; Dodge et al., 2001). Despite the identification of more than 50 different AKAPs in various cell types, with additional variations in the conformation and assembly of anchoring molecules (Zhu et al., 2019; He et al., 2024), they all share an ability to scaffold protein kinase A (PKA). The diversity of AKAPs allows for directing PKA and other signaling molecules to specific subcellular locations and microdomains where signaling events are localized (Wild and Dell'Acqua, 2018; Oliveria et al., 2007; Boczek and Kapiloff, 2020; Mackiewicz and Boczek, 2025; Tomczak et al., 2024). This spatial targeting of signaling machinery minimizes random crosstalk between signaling pathways and ensures that neuronal responses are finely tuned.

Previous studies have shown that mAKAP (AKAP6) is targeted to the perinuclear space in hippocampal neurons through its interaction with nesprin-1α (Michel et al., 2005; Boczek et al., 2019). This precise localization of the scaffold is crucial for the pro-survival response of injured retinal ganglion cells (RGCs) and hippocampal neurons (Wang et al., 2015; Boczek and Kapiloff, 2020). mAKAP is a scaffold protein that exists in two alternatively spliced forms: mAKAPα and mAKAPβ. The longer form (250 kDa), mAKAPα, is preferentially expressed in the brain, whereas the shorter one, mAKAPβ, which lacks the first 244 amino acids, is predominantly expressed in cardiac and skeletal myocytes (Michel et al., 2005; Pare et al., 2005a; Passariello et al., 2015). Besides cAMP, mAKAP binds a large number of enzymes involved in cell signaling, including the cAMP target Epac1, adenylyl cyclases (types II and V), the cAMP-specific phosphodiesterase 4D3, and the protein phosphatases calcineurin (CaN) and PP2A (Passariello et al., 2015). By binding to mAKAP, these signaling molecules can modulate the activity of transcription factors such as the myocyte enhancer factor-2 (MEF2) and the nuclear factor of activated T-cell (NFATc), which have been implicated in neuronal survival and differentiation (Lisek et al., 2023; Mackiewicz et al., 2023). Despite many signaling molecules being tethered by mAKAPα, its function in hippocampal neurons remains unclear. Our recent reports suggest that mAKAPα may also be required for activity-dependent axonal outgrowth (Boczek et al., 2021; Boczek et al., 2019), but no involved mechanism has been demonstrated so far.

In this study, we show that mAKAPα is essential for electrical activity-dependent axonal elongation. We also demonstrate that mAKAPα serves as a neuronal scaffold for perinuclear CaN/NFAT/MEF2 signaling. Moreover, we show that CaN and MEF2A, but not NFAT, tethering to the mAKAPα complex is required for both basal and activity-dependent axonal elongation. Our results illustrate how mAKAPα scaffold organizes CaN/MEF2 signaling, thus providing the molecular architecture for signal transduction involved in axon elongation.

## 2 Materials and methods

### 2.1 Reagents

Dulbecco’s Modified Eagle Medium (DMEM, Gibco™, UK), Horse Serum (HS, Biowest, France), Neurobasal (Gibco™, UK), B-27 supplement (Gibco™, UK), Insulin (Sigma-Aldrich, USA), Sodium pyruvate (Gibco™, UK), Glutamine (Biowest, France), Hanks’ Balanced Salt Solution (1X) (HBSS) without calcium and magnesium (GibcoTM, UK), Hanks’ Balanced Salt Solution (1X) (HBSS) with calcium and magnesium (GibcoTM, UK), Poly-L-lysine hydrobromide (Sigma-Aldrich, USA), Arabinosyl cytosine (Sigma-Aldrich, USA), Lipofectamine LTX Reagent (ThermoFisher, USA), Pierce™ RIPA buffer (ThermoFisher, USA), Bovine Serum Albumin (BSA, Millipore, USA), BCIP/NBT and ECL Western Blot System (Bio-Rad Laboratories, USA), Protein A/G PLUS-Agarose (Millipore, USA), EverBrite™ Hardset Mounting Medium (VWR, USA), FITC Annexin V Apoptosis Detection Kit (Sigma-Aldrich, USA), Trizol (Santa Cruz Biotechnology, USA), HOT FIREPol® EvaGreen® qPCR Mix Plus (Cytogen, Poland), Dual-Glo Luciferase Assay System (Promega, USA). NFAT luciferase reporter lentivirus and firefly luciferase lentivirus were sourced from BPS Bioscience (USA). Primers used in the Real Time PCR Reaction were provided by the BioCat GmbH (Germany).

### 2.2 Plasmids, viruses, and antibodies

Plasmids and lentiviral vectors were constructed by Genewiz (Germany) using the company’s proprietary techniques. The pmCherry-C1-CBD expression vector contains cDNA encoding a peptide corresponding to mAKAPα amino acids 1,286–1,345 (Calcineurin Binding Domain – “CBD”) fused to the C-terminus of mCherry. The pEGFP plasmid includes a fragment of the N-terminal mAKAPα domain that is not expressed in mAKAPβ (aa3-196) – GFP-(aa3-196), or a fragment of mAKAPα encoding the sequence for MEF2 transcription factor binding (aa301-500) – GFP-(aa301-500) inserted downstream of GFP. The mCherry plasmid (#176016, Addgene) and EGFP plasmid (#165830, Addgene) were used as backbones. NFATc4 was overexpressed using a ready-to-use pEGFP-C1 NFATc4 vector (#10961, Addgene). VIVIT peptide was expressed using GFP-VIVIT plasmid (#11106, Addgene). Endogenous MEF2A expression was silenced using MEF2A-shRNA plasmid (#32899, Addgene). Before transfection into primary neurons, plasmids were sequenced and expressed in HeLa or HEK293 cells.

A lentivirus expression system was used to deliver mAKAPα shRNA to the neuronal cultures. The sequences were as follows: scrambled shRNA: 5′-GACGAACCCCTGTTCCGAATT CAAGAGATTCGGAACAGGGGTTCGTCTTTTT-3′ and mAKAPα shRNA – 5′-GACGAACCTT CCTTCCGAATTCAAGAGATTCGGAAGGAAGGTTC GTCTTTTT-3′, as published in Pare et al. (2005b). Both vectors contain the U6 promoter and an mCherry coding sequence. The titer of both lentiviruses was ≥10×10^8^ TU/mL. Hippocampal neurons were infected with MOI 5 and cultured for 48 h in growth medium before further analysis. The efficiency of lentiviral-mediated silencing was tested in PC12 cells overexpressing full-length rat mAKAPα protein. PC12 cells were maintained and transfected as described previously (Boczek et al., 2017).

Adeno-associated virus serotype 2 (AAV2) used to express the GFP-tagged CBD domain of mAKAPα (rAAV-CAG-1286-1345 CBD-EGFPSV40 polyA) was constructed based on the mCherry-CBD plasmid and produced by BioHippo Inc. (USA). rAAV-CAG-EGFP was used as the control. The titer of both AAVs was ≥2.00×10^12^ vg/mL. Hippocampal neurons were transduced with MOI 1000 and cultured for 48 h in growth medium before experiments. The antibodies used in this study are listed in Table 1.

**Table 1:** Antibodies used in this study.

Antibody/ANTIGEN	Species, catalog #	Company
Alexa Fluor 488 anti-rabbit	Chicken, A-21441	ThermoFisher
Alexa Fluor 594, anti-mouse	Goat, A-11020	ThermoFisher
Anti-goat AP	Rabbit, NBP1-74826 polyclonal	Novusbio
Anti-goat HRP	Mouse, sc-2354	Santa-Cruz Biology
Anti-mouse AP	Goat, A-3562 polyclonal	Sigma -Aldrich
Anti-mouse HRP	Goat, AS003	ABclonal
Anti-rabbit AP	Mouse, A2306 monoclonal	Sigma -Aldrich
Anti-rabbit HRP	Goat, AS014	ABclonal
Bcl2	Rabbit, ab196495 polyclonal	Abcam
GAPDH	Mouse, G8795 monoclonal	Sigma -Aldrich
iNOS	Rabbit, A3774 monoclonal	ABclonal
mAKAP	Goat, NB300-869 polyclonal	Novusbio
MEF2A	Rabbit, A12059 polyclonal	ABclonal
Mif	Rabbit, A22623 monoclonal	ABclonal
Nfatc4	Rabbit, AV32715 polyclonal	Sigma -Aldrich
Phospho-Nfatc4 (ser289)	Rabbit, PA-5105650 polyclonal	ThermoFisher
CaNAα	Rabbit, sc-9070 polyclonal	Santa-Cruz Biology
Prkar1a	Rabbit, A19334, polyclonal	ABclonal
Tpa/Plat	Rabbit, A4210 monoclonal	ABclonal
GFP	Mouse, MA5-15256 monoclonal	Invitrogen
Flag	Rabbit, F7425 polyclonal	Merck

### 2.3 Isolation and culture of primary rat hippocampal neurons

All procedures for animal handling were approved by the Institutional Animal Care and Use Committee at the Medical University of Lodz. Hippocampal neurons were isolated from embryonic day 17 (E17) Sprague–Dawley rat embryos of either sex. In brief, hemispheres were dissected in PBS medium on ice and digested with 0.05% trypsin–EDTA in HBSS without calcium and magnesium for 30 min at 37°C. The separated tissues were centrifuged at 250 × g for 2 min and then triturated with a fire-polished glass pipette in HBSS with calcium and magnesium. The dissociated neurons were seeded on nitric acid-treated 25 mm cover glasses coated with poly-L-lysine in plating medium (10% v/v horse serum in DMEM). Four hours after plating, the medium was replaced with maintenance Neurobasal defined medium supplemented with 2% B27, 1 mM glutamine, 1 mM sodium pyruvate, and 5 μg/mL insulin. On DIV3, 4 μM arabinosyl cytosine was added to prevent glial proliferation. To extend cultures beyond 4 days, half of the medium was exchanged with fresh medium on either DIV3 or DIV4.

### 2.4 Axon extension assay

For the neurite extension assay, on DIV3-4, hippocampal neurons were transfected or co-transfected with appropriate plasmids using Lipofectamine LTX with Plus Reagent, or transduced with lentiviruses. To promote neurite elongation, 30 mM KCl was added directly to the growth medium. Cell imaging was performed 24–48 h after transfection/transduction. Images were acquired on a Leica DMi8 inverted microscope, and the longest neurite per cell (~20 cells on average in each experiment) was measured using ImageJ with the Simple Neurite Tracer plugin.

### 2.5 Western blotting

Cells were lysed using RIPA buffer supplemented with a protease inhibitor cocktail. Following centrifugation, total protein lysate was quantified colorimetrically with the Bio-Rad Protein Assay Kit. Next, 10–50 μg of the protein samples were fractionated in 8% or 10% polyacrylamide gels and transferred to a nitrocellulose membrane using a semi-dry method. The membranes were immersed in blocking buffer (5% BSA) in TBS-T (10 mM Tris–HCl, pH 7.4, 150 mM NaCl, and 0.05% Tween-20) for 1 h at room temperature and incubated with primary antibodies for 24 h at 4°C. The primary antibodies used are as follows: Tpa/Plat (1:1000), Mif (1:1000), iNOS (1:1000), Prkar1a (1:1000), and GAPDH (1:1000). Following three washes in TBS-T buffer, the membrane was probed with secondary antibodies conjugated with alkaline phosphatase (AP) (1:5000) for 2 h or conjugated with horseradish peroxidase (HRP) (1:20,000) for 1 h at room temperature. BCIP/NBT or ECL Western Blot System was used to detect immunoreactive bands. The membranes were scanned densitometrically, and the optical density of the bands was quantified using ImageJ. The results are expressed as arbitrary units after normalization to the endogenous GAPDH level, providing a quantitative assessment of protein expression levels.

### 2.6 Immunoprecipitation

For the co-immunoprecipitation assay, cells were treated with 30 mM KCl for 20 min. Additionally, to inhibit calcineurin activity, neurons were treated with 5 μM cyclosporine (CsA), and 1 mM ethylenediaminetetraacetic acid (EDTA) was used to chelate Ca^2+^. Next, 200 μg of proteins was incubated with anti-mAKAPα antibodies (~2 μg of antibodies/200 μg of lysate proteins) overnight at 4°C, followed by incubation with 25 μL of Protein A/G PLUS-Agarose beads at 4°C for 2 h. The immunocomplexes were recovered by centrifugation at 8130 x g for 5 min, washed three times with PBS, eluted with 60 μL of SDS-PAGE sample buffer (62.5 mM Tris–HCl, pH 6.8, 10% glycerol, 2% SDS, and 0.001% bromophenol blue) containing 5% *β*-mercaptoethanol, and subjected to immunoblotting. The membranes with immunoprecipitated mAKAPα protein were probed with anti-NFATc4 (1:500), anti-phospho-NFATc4 (Ser289) (1:1000), anti-CaNAα (1:500), and MEF2A (1:1000) antibodies. For interaction in heterologous cells, aa3-196 peptide was immunoprecipitated with anti-GFP antibody and immunoblotted with anti-Flag antibody (1:1000). Next, immunoblots were incubated with secondary antibodies (1:5000 or 1:20,000) conjugated to AP or HRP. Bands were visualized using the AP system or ECL substrates (Bio-Rad, USA) following the manufacturer’s protocol. The results were normalized to OD/mg protein and are presented as the fold change in relation to control cells.

### 2.7 Immunocytochemistry

Isolated primary neurons were transfected with mCherry or mCherry-CBD and treated with 30 mM KCl for 24 h. For immunostaining, a day after transfection, cells were washed three times with cold PBS and fixed in 4% PFA for 10 min at room temperature. After several washes with PBS, neurons were permeabilized with Triton X-100 and immersed in blocking buffer (1% BSA in PBS-T) for 1 h at room temperature. Subsequently, cells were incubated overnight at 4°C with a primary anti-Bcl2 antibody (1:200) in 1% BSA in PBS-T. Following washing with PBS, neuronal cells were incubated with secondary antibodies conjugated to Alexa Fluor 488 (1:1000) for 2 h at room temperature. Next, coverslips with hippocampal neurons were mounted in mounting medium on glass slides. Images of transfected cells and immunolabeled Bcl-2 were acquired on a Leica DMi8 inverted microscope. Fluorescence intensity was measured using ImageJ.

For Annexin V staining, 24 h after transfection, neurons were washed two times with PBS and 1X Binding Buffer (FITC Annexin V Apoptosis Detection Kit I). Next, cells were incubated with Annexin V diluted 1:100 in 1X Binding Buffer for 15 min at room temperature in the dark. After incubation, images of positive transfected neurons were acquired on a Leica DMi8 inverted microscope. Fluorescence intensity was measured using ImageJ.

### 2.8 Total RNA isolation, real time PCR, and microarrays screening

Total RNA was extracted from the isolated hippocampal neurons using Trizol reagent according to the manual provided by the manufacturer. Single-stranded cDNA was synthesized from 1 μg of isolated RNA and oligo (dT) primers with M-MLV reverse transcriptase. The Real-time PCR conditions included an initial denaturation at 95°C for 15 min, followed by 40 cycles at 95°C for 15 s, 60°C for 30 s and 72°C for 30 s using the Abi Prism 7,000 sequence detection system using Eva Green Master Mix. Primers used in the reactions: Akap6 set#1 (NM_022618), Akap6 set#2 (NM_022618), Akap6 set#3 (NM_022618). The specificity of the product was assessed by agarose gel electrophoresis.

For microarray screening, total RNA was extracted from the primary neurons 48 h after transduction with AAV2-GFP or AAV2-GFP-CBD, using Trizol Reagent according to the manual provided by the manufacturer. cDNA amplified from 2 μg of neuronal RNA was hybridized with RT^2^ Profiler™ PCR Array Rat calcium/cAMP signaling, and the reaction was performed using HOT FIREPol® EvaGreen® qPCR Mix Plus. Real-time PCR reactions were carried out under the following conditions: an initial cycle at 95°C for 10 min, followed by cycles at 95°C for 15 s, 60°C for 1 min, and a dissociation curve at 95°C for 1 min, 55°C for 30 s, and 95°C for 30 s. The fold change was calculated by a method of Livak and Schmittgen (2001). Data were analyzed using Qiagen PCR Array Data Analysis Web Portal. The microarray analysis was run in triplicate, and the RT^2^ software averaged the triplicate normalized expression for each gene (ΔCt) before calculating ΔΔCt between the control (AAV2-GFP) and experimental group (AAV2-GFP-CBD). Housekeeping genes used for normalization were selected based on the recommendations of Vandesompele et al. (2002). Two Microarray Quality Control studies demonstrated that a *p*-value calculation based on fold change could be considered sufficient for obtaining reproducible results across microarray analyses, including RT^2^ Profiler™ PCR Arrays (Shi et al., 2006; Shi et al., 2010).

### 2.9 *In vitro* luciferase reporter assay

NFAT transcriptional activity was done essentially as described previously (Na et al., 2010; Mackiewicz et al., 2024) with some modifications. Briefly, lentiviral particles were engineered to carry a firefly luciferase gene under the control of the NFAT response element positioned upstream of the minimal TATA promoter. Primary hippocampal neurons were transduced with Lenti-NFAT luciferase reporter and Lenti-luciferase at DIV0, and the neurons were cultured for 3 days. NFAT transcriptional activity in control GFP-expressing cells or GFP-(aa3-196)-expressing cells was measured in cell lysates using the Dual-Glo Luciferase Assay System (Promega, USA) according to the manufacturer’s instructions. The expression of the NFAT luciferase reporter was normalized to the expression of firefly luciferase. The fold increase of normalized NFAT luciferase reporter expression was then calculated relative to the baseline values. Ca^2+^-dependent increase in NFAT transcriptional activity was determined following a 20-min stimulation with 30 mM KCl. CsA (10 μM) was added 1 h prior the experiment.

### 2.10 Statistics

Statistical analysis was conducted using GraphPad Prism version 8.0.1. Single comparisons were done using a two-tailed Student’s t-test. All data involving multiple comparisons were analyzed using one- or two-way ANOVA with post-hoc correction. All datasets are expressed as means of at least three independent experiments with standard error of the mean (SEM). Significance is represented as follows: *p < 0.05, **p < 0.01, ***p < 0.001.

## 3 Results

### 3.1 mAKAPα is essential for KCl-dependent extension of primary hippocampal neurons

**Figure 1:**
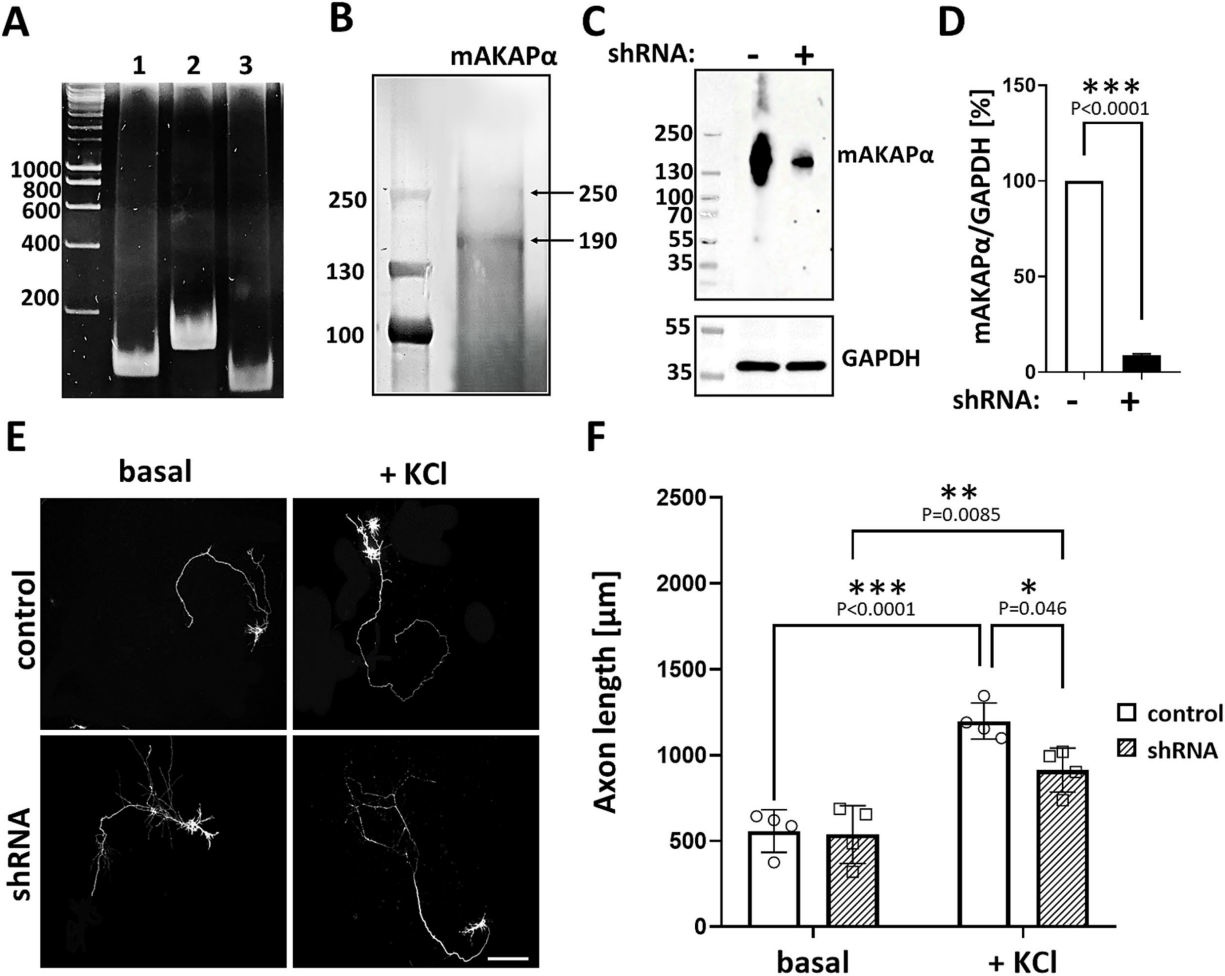
mAKAPα silencing attenuated KCl-dependent neurite extension. (A) Representative photograph of agarose gel electrophoresis showing mAKAPα expression in primary rat hippocampal neurons. Lines 1–3 – the presence of mAKAPα mRNA demonstrated using three different primers. (B) Representative immunoblotting of mAKAPα. The immunoreactive bands were visualized using the BCIP/NBT alkaline phosphatase substrate. (C) The representative micrograph of mAKAPα knockdown with a lentivirus-based shRNA in heterologous expression system. PC12 cells were transfected with full-length rat mAKAPα and were subsequently transduced with either control (shRNA-) or mAKAPα-Lenti-shRNA (shRNA+) and the efficiency of silencing was evaluated 2 days after. GAPDH was used as a loading control. The bands were developed using chemiluminescent detection system. (D) Quantification of knockdown efficiency, normalized to GAPDH protein level. The results are presented as percentage change in mAKAPα protein level relative to scrambled shRNA-transfected cells, with expression in these cells set at 100%. Data from three independent experiments (*n* = 3). (E) Representative grayscale images of mCherry fluorescence for hippocampal neurons transduced with control or mAKAPα-Lenti-shRNA-mCherry virus and cultured in growth media containing 5 mM KCl (basal) or 30 mM KCl for 2 days. Scale bar 100 μm. (F) Quantification of axon length. Mean length of the longest neurite are shown for four independent experiments. *p < 0.05, **p < 0.01, ***p < 0.001.

Previous studies have indicated that mAKAPα is expressed in the brain, including the retina (Wang et al., 2015; Michel et al., 2005). Consistent with our earlier report (Boczek et al., 2019), we confirmed mAKAPα expression in primary rat hippocampal neurons isolated from E18 embryos. The presence of mAKAPα mRNA was detected with different sets of primers, producing PCR products between 111 bp and 163 bp (Figure 1A). Additionally, mAKAPα was immunoblotted in extracts prepared from isolated primary neurons (Figure 1B). The antibody raised against the LTMSVTLSPLRSQ sequence, corresponding to the N-terminus of mAKAPα (NP_004265.3) which is absent in mAKAPβ, detected a band around 250 kDa, similar to the previously reported mAKAPα mobility in the brain (Wang et al., 2015). A band of ~190 kDa was also detected in the neuronal extract, consistent with the size declared by the manufacturer. However, since the ~190 kDa band is smaller than the molecular weight of mAKAPα, this protein is likely a degradation product of the 250 kDa mAKAPα.

Given that mAKAPα is indispensable for RGC survival and outgrowth *in vitro* (Wang et al., 2015), we evaluated whether it is also required for KCl-mediated elongation of hippocampal neurons. To address this, hippocampal neurons were transduced with lentivirus carrying shRNA against mAKAPα in the presence or absence of KCl (30 mM), which has been shown to induce neuronal outgrowth in a cAMP- and PKA-dependent manner (Goldberg et al., 2002). The specificity and efficiency of shRNA were evaluated in our previous paper, demonstrating compromised cAMP synthesis in the perinuclear space in the absence of the scaffold (Boczek et al., 2019). Two days after transduction, the mAKAPα protein level decreased by ~90% relative to the scrambled Lenti-shRNA control group (Figures 1C,D). Following mAKAPα depletion, the mean length of the longest neurite per cell was markedly less compared to the control (Figures 1E,F) in depolarizing conditions. No effect of mAKAPα downregulation on axonal length was observed in resting conditions (5 mM KCl) (Figures 1E,F). Together, these data indicate that mAKAPα is required for depolarization-mediated axonal elongation but is dispensible for basal neuronal outgrowth.

### 3.2 mAKAPα is a scaffold for perinuclear CaN/NFAT signaling

**Figure 2:**
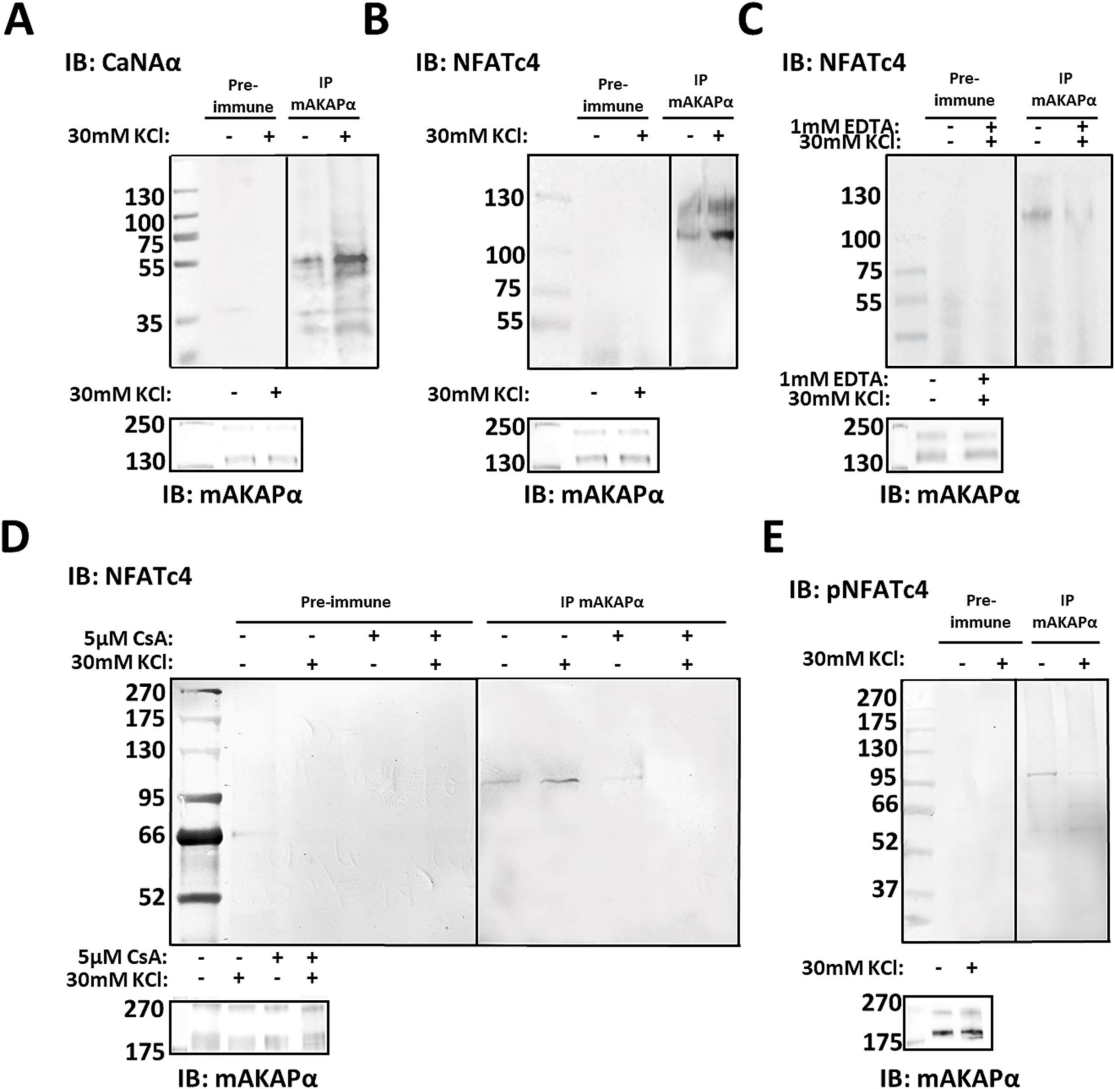
CaNAα and NFATc4 tethering to mAKAPα signaling complex is enhanced by neuronal activity. (A,B) The mAKAPα antibody was used for immunoprecipitation of endogenous calcineurin (catalytic Aα subunit, CaNAα) or NFATc4 from primary rat hippocampal neurons in a steady-state conditions and following 20-min depolarization with 30 mM KCl. (C) mAKAPα-NFATc4 interaction in the presence of 1 mM EDTA. (D) mAKAPα -NFATc4 binding in the presence of calcineurin inhibitor-cyclosporine A (CsA). (E) Co-immunoprecipitation of phosphorylated NFATc4 (S289) with mAKAPα following neuronal depolarization with 30 mM KCl. All blots shown are representative of experiments repeated at least three times.

Having shown that mAKAPα function is relevant to activity-dependent neuronal elongation, we investigated the mAKAPα-regulated signaling that underlies the attenuated KCl effect. The Ca^2+^-activated protein phosphatase calcineurin (CaN) has previously been demonstrated to interact with mAKAP in cardiac myocytes (Pare et al., 2005b; Li et al., 2010). To determine whether this interaction occurs in primary hippocampal neurons, we probed mAKAPα immunoprecipitates for CaN by using an antibody that predominantly recognizes the catalytic subunit of calcineurin Aα (CaNAα). A significant amount of CaNAα co-immunoprecipitated with mAKAPα in a steady-state conditions (Figure 2A). Further experiments were performed to evaluate whether neuronal depolarization can affect CaNAα/mAKAPα association. Co-immunoprecipitation of both proteins following a 20-min depolarization with 30 mM KCl increased CaNAα immunoreactivity in mAKAPα immunoprecipitates, suggesting that neuronal activity enhances CaN tethering to the scaffold.

Based on this observation, we aimed to test for potential CaN substrates that might also dock on the mAKAPα scaffold. Our initial approach involved NFATc4 protein, a well-known downstream target of CaN involve in neurotrophin-mediated survival of hippocampal neurons (Groth and Mermelstein, 2003). To determine whether mAKAPα and NFATc4 interact in hippocampal neurons, mAKAPα immunocomplexes were probed for co-immunoprecipitation of NFATc4. Western blot analysis demonstrated that NFATc4 binding to the mAKAPα signalosome was enhanced following KCl depolarization (Figure 2B) and blocked in the presence of extracellular Ca^2+^ chelators, i.e., EDTA (Figure 2C).

Given that both CaNAα and NFATc4 bound more strongly to the scaffold in the presence of Ca^2+^, we suspected that CaNAα may retain its catalytic activity within the complex and regulate NFATc4 binding. mAKAPα/NFATc4 co-immunoprecipitations were repeated in the presence of cyclosporin A – a calcineurin inhibitor, demonstrating a significant decrease in the protein–protein interaction with the inhibitor (Figure 2D). We anticipated that increased neuronal activity recruits active CaNAα to the scaffold, which dephosphorylates bound NFATc4. Following KCl treatment, phosphorylated NFATc4 (S289) co-immunoprecipitated less with mAKAPα (Figure 2E), supporting a decreased phosphorylation state of NFATc4 with the scaffold.

### 3.3 CaN association with mAKAPα is required for depolarization-induced neuronal outgrowth

**Figure 3:**
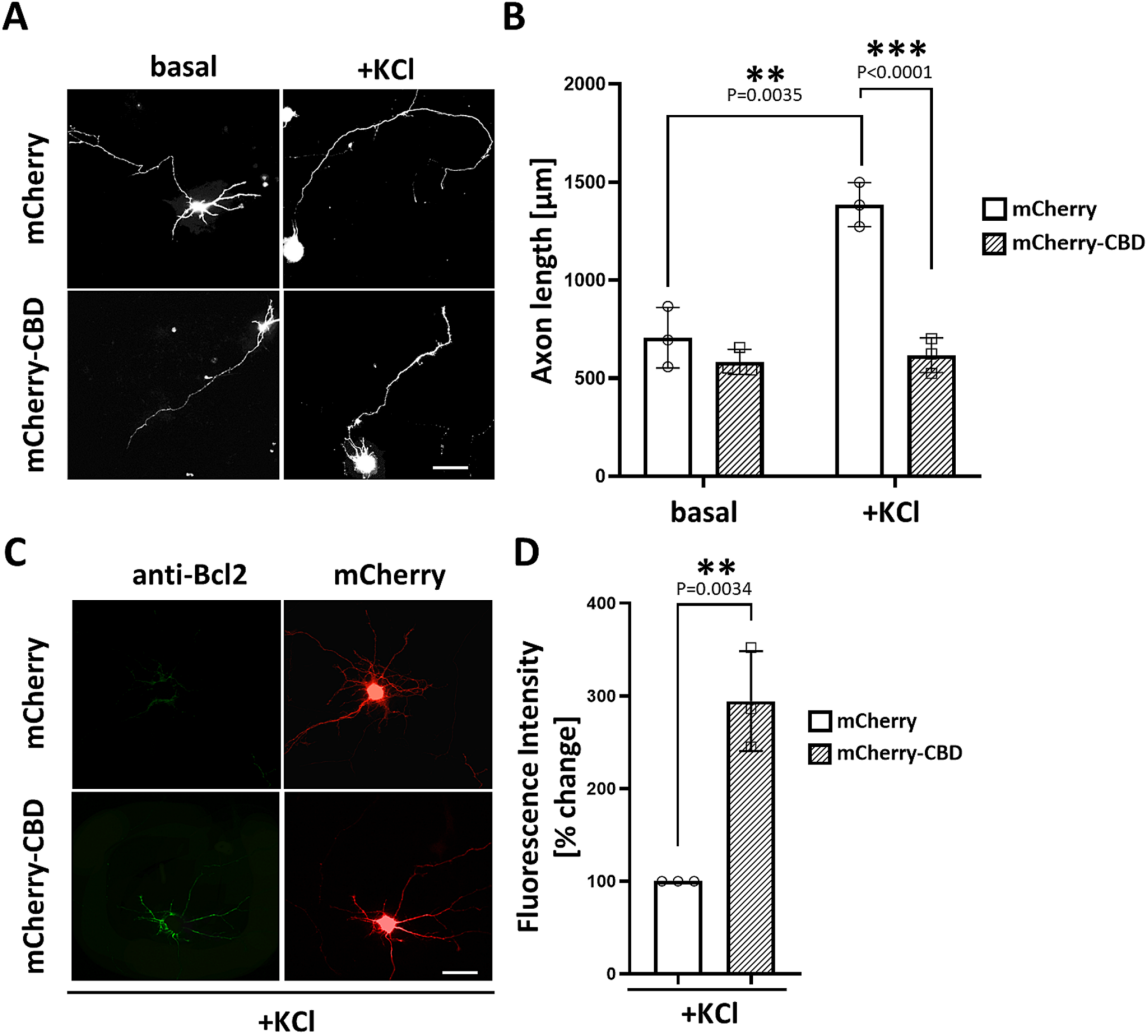
Requirement of CaN association with mAKAPα for KCl-induced neuronal outgrowth. (A) Representative grayscale images of hippocampal neurons expressing mCherry or calcineurin-displacing peptide (mCherry-CBD). Neurons were transfected with appropriate plasmids on DIV3-4. After transfection, 30 mM KCl was added to the medium, and neurons were assayed for axonal outgrowth 24 h later. Scale bar: 100 μm. (B) Quantification of axon length. Mean length of the longest neurite are shown for three independent experiments. (C) Representative grayscale images of neurons expressing mCherry or mCherry-CBD stained for Bcl-2. Scale bar: 100 μm. (D) Quantification of Bcl-2 fluorescence intensity. The results are expressed as percentage changes relative to mCherry-expressing neurons, with average fluorescence in these neurons set at 100%. The fluorescence was measured in three independent experiments (10–15 cells were measured in each experiment) using ImageJ software. Individual points on the graph represent an average fluorescence from each experiment. **p < 0.01, ***p < 0.001.

Previous studies have demonstrated that CaNAβ binds directly to a unique site within mAKAP (residues 1,286–1,345), which is both necessary and sufficient for phosphatase binding (Dodge-Kafka et al., 2005; Pare et al., 2005b). To address the significance of CaN scaffolding by mAKAPα for axonal outgrowth, we generated a genetically encoded anchoring disruptor by fusing residues 1,286–1,345 of mAKAPα (referred to here as the Calcineurin Binding Domain – “CBD”) to the C-terminus of mCherry. Hippocampal neurons were transfected with expression plasmids for either mCherry or mCherry-CBD, cultured for 24 h in the presence or absence of KCl, and assayed for neurite extension. Consistent with the slower axon growth of mAKAPα-depleted neurons (see Figure 1F), mCherry-CBD expression abolished KCl-dependent neuronal extension compared to mCherry-expressing neurons (Figures 3A,B). These findings indicate that depolarization-induced CaN recruitment to the mAKAPα scaffold is required for axonal outgrowth during chronic KCl exposure.

**Figure 4:**
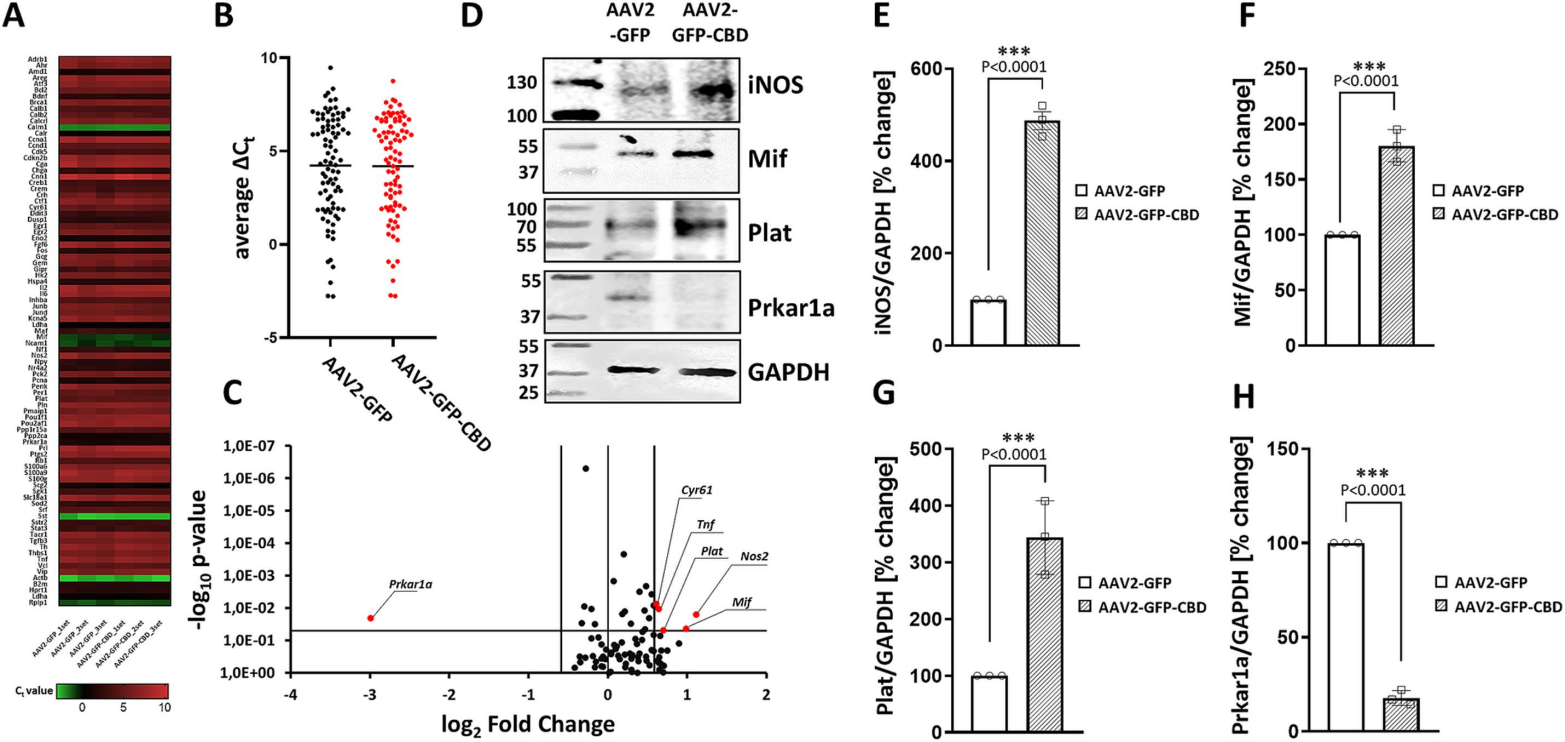
The effect of CaN anchoring disruptor on gene expression involved in cAMP/calcium signaling. (A) Rat transcription factors qPCR microarray was used to assess expression changes of transcription factors in hippocampal neurons. Data are presented as a heat map, adjusted to reflect variations from minimum to maximum for individual runs. The map was generated using GraphPad Prism software based on the expression data. (B) Dot plot comparison of average C_t_ values of analyzed genes. The average is presented as a black horizontal line. (C) Volcano plot analysis of the gene expression profile. Fold difference in AAV2-GFP-CBD neurons was calculated in relation to AAV2-GFP control using the comparative 2^-∆∆Ct^ method. The results were divided into two groups considering a 1.5-fold change as the minimum. Black dots: fold change <1.5 or *p* > 0.05; red: fold change >1.5 and *p* < 0.05. (D) Representative Western blot analysis of iNOS, Mif, Prkar1a, and Plat levels after disruption of CaN-mAKAP interaction using AAV2-GFP-CBD compared to the control AAV2-GFP. GAPDH was used as a loading control. Full blots can be found in Supplementary Figure S2. (E–H) Quantification of protein levels after normalization to the GAPDH loading control. The results are presented as percentage changes relative to AAV2-GFP transduced neurons, with the average fluorescence in these cells set at 100%, *n* = 3 independent experiments. ***p < 0.001. CBD, calcineurin-displacing peptide.

To understand the phenomenon of inhibited axon growth in the presence of mCherry-CBD under stimulated conditions, we tested whether CaN displacement may result in neuronal apoptosis. Staining with annexin V, which detects the apoptosis-related shift of phosphatidylserine to the outer leaflet of the plasma membrane, revealed no significant differences between mCherry- and mCherry-CBD-expressing neurons (Supplementary Figure S1). Interestingly, immunocytochemical staining of Bcl-2, a member of the anti-apoptotic protein family, showed increased expression of this protein in neurons expressing mCherry-CBD compared to the control (Figures 4C,D), suggesting a plausible adaptive response to potentially counterbalance apoptotic signaling.

### 3.4 Disruption of CaN/mAKAPα interaction causes changes in signal transduction

Based our previous work demonstrating that mAKAPα selectively coordinates Ca^2+^-dependent cAMP signaling for axonal outgrowth *in vitro* and neuroprotection *in vivo* (Boczek and Kapiloff, 2020; Mackiewicz and Boczek, 2025; Tomczak et al., 2024; Boczek et al., 2019), we hypothesized that the loss of KCl effect on axon elongation in the presence of the mCherry-CBD peptide could be due to peptide-related deficits in signal transduction and gene expression. To explore this possibility, hippocampal neurons were transduced with AAV2 for GFP-CBD or GFP control. A day after transfection, 30 mM KCl was added, and neurons were chronically depolarized for another 24 h. Microarray screening of genes involved in cAMP/Ca^2+^ signaling (the full list of genes can be found in Supplementary Table S1) showed no significant changes in the average Ct values between AAV2-GFP-CBD and AAV2-GFP control (Figures 4A,B), suggesting that displacing CaN from the mAKAPα signalosome does not affect global gene expression. Considering a 1.5-fold change as a minimum and *p* < 0.05, we identified five genes with significantly upregulated expression: *Nos2*, *Mif*, *Plat*, *Tnf*, *Cyr61* and one with markedly downregulated expression: *Prkar1a* (Figure 4C). We further confirmed that AAV2-GFP-CBD treatment affected the levels of four of the corresponding proteins (Figures 4D–H).

### 3.5 N-terminus of mAKAPα is sufficient for NFATc4 binding but is not required for axonal outgrowth

**Figure 5:**
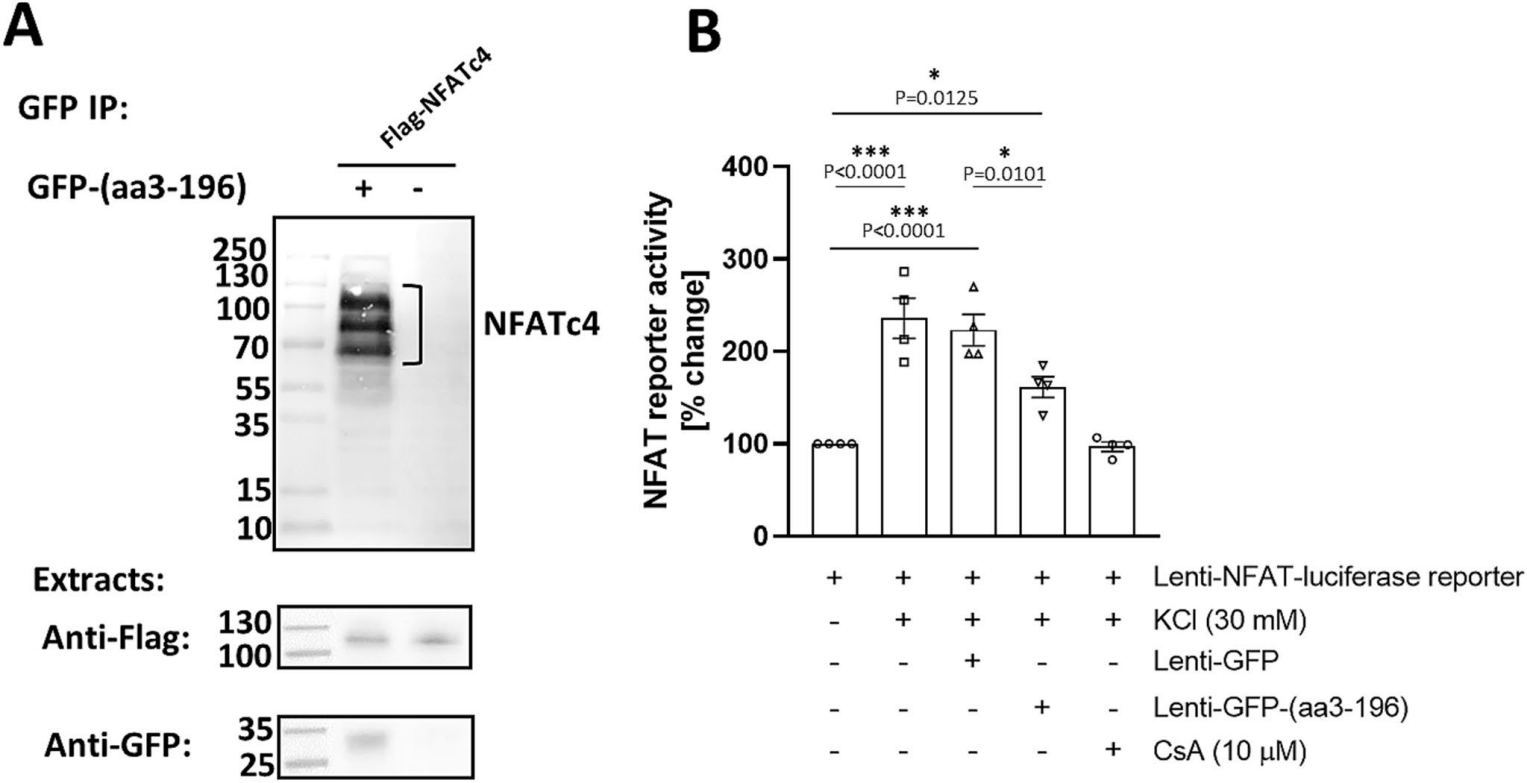
NFATc4 interacts with N-terminal domain of mAKAPα. (A) COS-7 cells were transfected with GFP-tagged peptide corresponding to 3–196 amino acid sequence of mAKAPα and Flag-tagged NFATc4. Two days later, proteins in the immunoprecipitates (top panel) and in total extracts (bottom panels) were detected using GFP- and Flag- specific antibodies. (B) Primary hippocampal neurons were transduced with an NFAT dual-reporter lentivirus and other viruses as indicated in the graph on DIV0 and cultured until DIV3. NFAT transcriptional activity was assessed in cell lysates by measuring luciferase activity (*n* = 4). The results are expressed as percentage changes in fold induction relative to baseline activity, which is set at 100%. KCl (30 mM) was applied for 20 min. Cyclosporin A (CsA, 10 μM) was added 1 h prior the experiment. *p < 0.05, ***p < 0.001.

Given our new findings that neuronal activity promotes both CaN and NFATc4 tethering to mAKAPα, we asked whether inhibition of NFAT binding would also affect neurite outgrowth. Considering the difference in the N-terminal domain between mAKAPα and *β* isoforms, we constructed a GFP-tagged peptide corresponding to amino acids 3–196 of mAKAPα. Expression of this peptide is expected to displace NFAT from the N-terminus of mAKAPα. The GFP-(aa3-196) and Flag-NFATc4 were co-expressed in COS-7 cells and analyzed by co-immunoprecipitation. Immunoprecipitation using anti-GFP antibodies resulted in co-purification of NFATc4 (Figure 5A).

We next tested the ability of the GFP-(aa3-196) peptide to regulate NFAT transcriptional activity in primary hippocampal neurons using a transient luciferase reporter assay (Figure 5B). To ensure effective expression, we constructed a Lenti-GFP-(aa3-196) vector and used lentiviral particles to deliver it along with a Lenti-NFAT luciferase reporter. As expected, stimulation with 30 mM KCl resulted in a 3.1-fold increase in NFAT transcriptional activity, which was completely inhibited by cyclosporine A. Remarkably, expression of GFP-(aa3-196) decreased NFAT activity by ~40% compared to neurons expressing GFP only. These results indicate that N-terminus of mAKAPα binds NFAT to regulate its transcriptional activity in response to neuronal depolarization. This raises the possibility that NFATc4 may be downstream of CaN/mAKAPα signaling in activity-dependent neuronal outgrowth.

**Figure 6:**
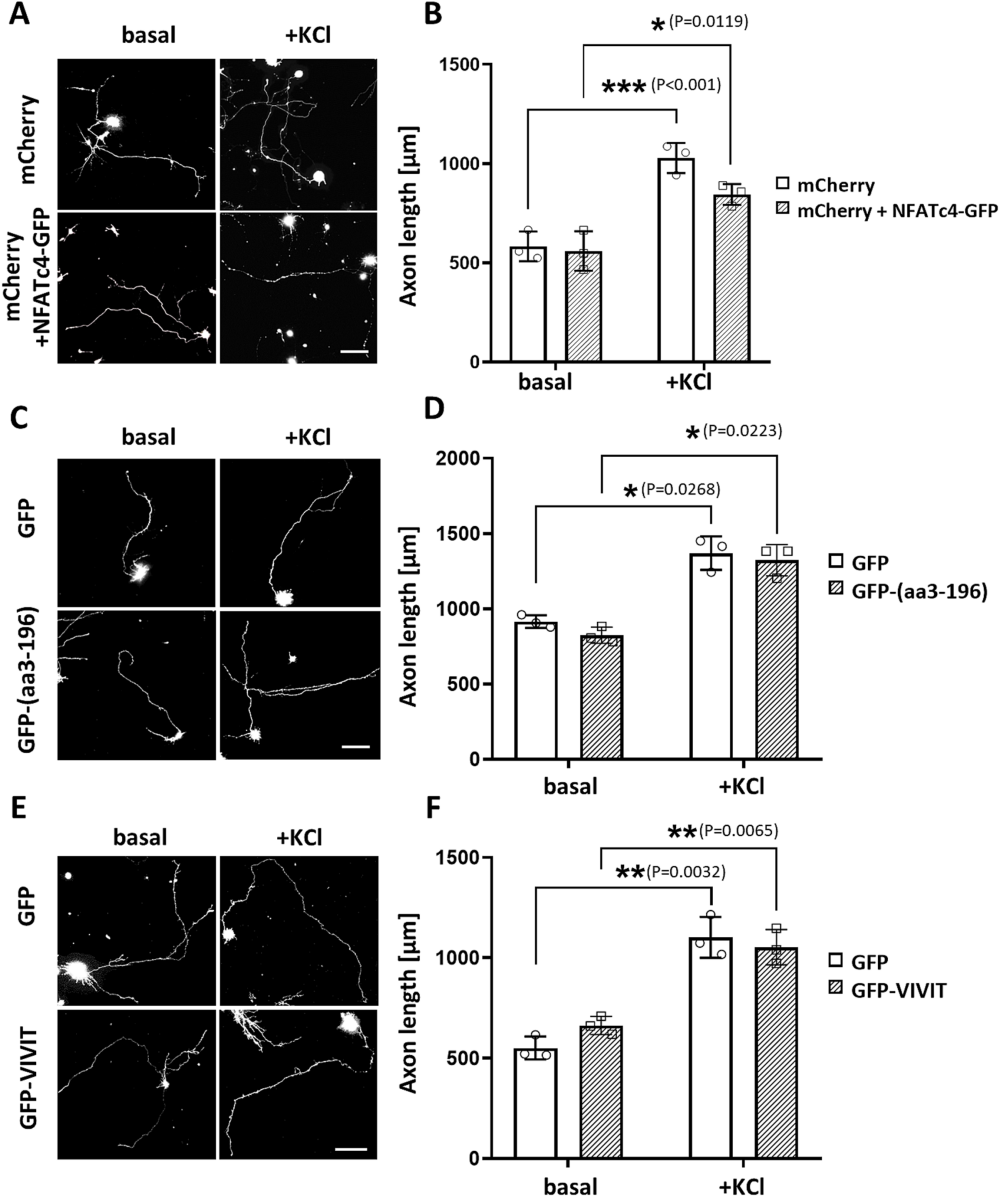
NFAT does not participate in axonal elongation of hippocampal neurons. (A) Representative grayscale images of mCherry fluorescence for hippocampal neurons transfected with mCherry only or mCherry along with NFATc4-GFP and cultured in a growth media containing 5 mM KCl (basal) or 30 mM KCl (+KCl) for 24 h. Scale bar 100 μm. (B) Quantification of axon length. Mean length of the longest neurite are shown for three independent experiments. (C) Representative grayscale images of hippocampal neurons transfected with GFP (control) or GFP-3-196 peptide. Scale bar 150 μm. (D) Quantification of axon length. The length of the longest neurite was measured, *n* = 3. (E) Representative grayscale images of neurons expressing GFP (control) or GFP-VIVIT. Scale bar 100 μm. (F) Quantification of axon length. The length of the longest neurite was measured, *n* = 3. *p < 0.05, **p < 0.01, ***p < 0.001.

To explore this hypothesis, we first overexpressed NFATc4-GFP and cultured hippocampal neurons in the absence or presence of 30 mM KCl for 24 h. We did not observe any effects on neuronal outgrowth under either basal or stimulated conditions (Figures 6A,B). Next, we assessed the relevance of the NFATc4/mAKAPα interaction to the inhibitory effect of the CaN-delocalizing peptide on neuronal outgrowth (Figures 6C,D). However, in contrast to the mCherry-CBD peptide (see Figures 3A,B), the expression of the GFP-(aa3-196) peptide did not affect axonal length, indicating a mechanism independent of NFAT binding to the N-terminal mAKAPα domain.

Because the GFP-(aa3-196) peptide only partially inhibited NFAT transcriptional activity, suggesting the possibility of multiple NFAT binding sites on mAKAPα, we transfected neurons with GFP-VIVIT (Figures 6E,F). VIVIT is a selective inhibitor of CaN-mediated dephosphorylation of NFAT that does not disrupt other CaN-dependent pathways. The efficacy of VIVIT in blocking NFAT activation is comparable to that of CsA, as we previously demonstrated in hippocampal neurons (Mackiewicz et al., 2024). The axon length of neurons expressing GFP-VIVIT was similar to that of neurons expressing GFP alone, indicating that NFAT activity is dispensable for hippocampal neuron growth *in vitro*.

### 3.6 KCl-induced neuronal outgrowth is mediated by mAKAPα-MEF2 signaling

**Figure 7:**
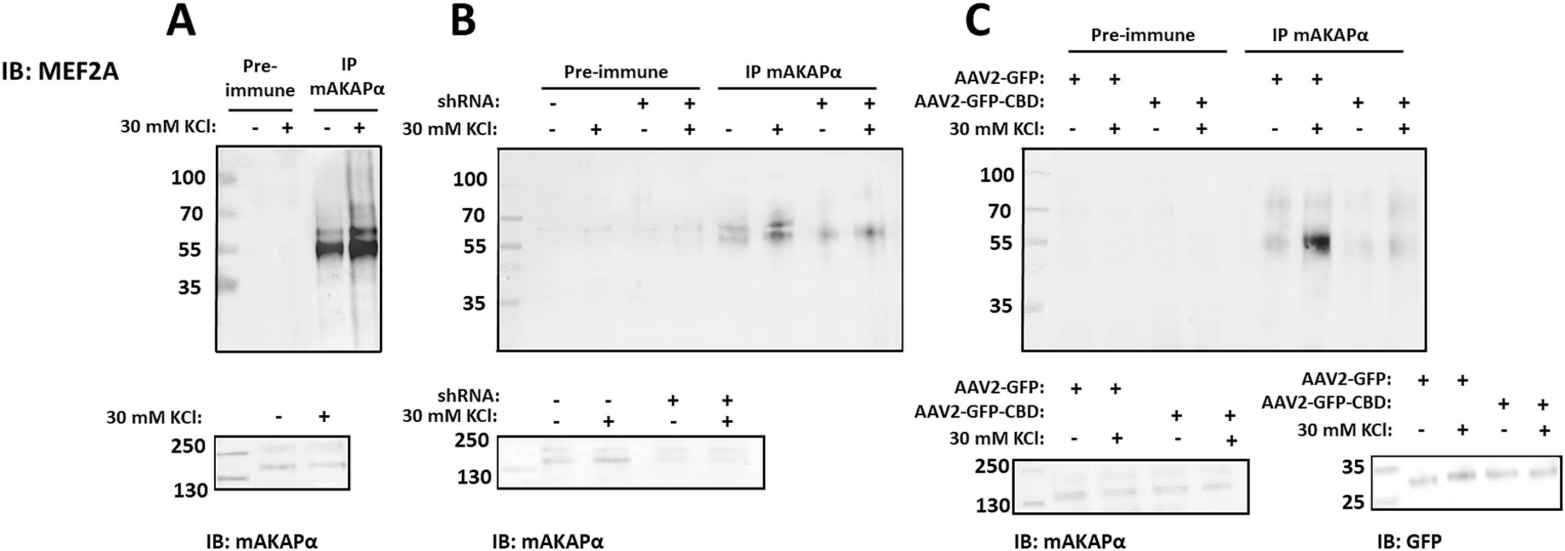
Activity-dependent MEF2A association with mAKAPα is regulated by CaN binding. (A) Endogenous MEF2A was immunoprecipitated using an mAKAPα antibody before and after stimulation with 30 mM KCl for 20 min. (B) mAKAPα expression was knocked down using a lentivirus-based shRNA, and mAKAPα immunocomplexes were probed for MEF2A 2 days later. (C) Hippocampal neurons were transduced with AAV2-GFP-CBD or AAV2-GFP. Two days post-infection, protein complexes were immunoprecipitated using an anti-mAKAPα antibody. Proteins in the immunoprecipitates and total extracts were detected using MEF2A- or mAKAPα-specific antibodies. GFP-CBD competes with CaN for binding to mAKAPα, but not with MEF2. All blots shown are representative of experiments repeated at least three times.

Another transcription factor which activation is controlled by CaN and it is tethered to mAKAPα signalosome is MEF2. Moreover, a growing body of evidence clearly indicates that MEF2 plays a critical role in neuronal development (Lisek et al., 2023). To determine whether mAKAPα and MEF2 interact in hippocampal neurons, mAKAPα immunocomplexes were probed for co-immunoprecipitation of MEF2A. Similar to CaN and NFATc4, Western blot analysis showed that MEF2A binds to the mAKAPα signalosome in a Ca^2+^-dependent manner (Figure 7A).

To determine whether mAKAPα is the scaffold required for the recruitment of MEF2A to the perinuclear compartment, MEF2A was immunoblotted in mAKAPα immunocomplexes from hippocampal neurons transduced with either control or mAKAPα shRNA (Figure 7B). Endogenous mAKAPα strongly co-immunoprecipitated with the MEF2A antibody only in cells expressing the scaffold. Furthermore, the KCl-enhanced MEF2A/mAKAPα association was significantly reduced in neurons expressing mAKAPα shRNA.

We next used the AAV2-GFP-CBD virus, which disrupts the full-length mAKAPα/CaN interaction, to assess whether CaN binding to the scaffold is a prerequisite for MEF2A association. The CBD peptide does not compete with MEF2 binding, as MEF2 and CaN bind to different mAKAPα fragments. As shown in Figure 7C, the binding of MEF2A to mAKAPα, both in the presence and absence of KCl, was reduced when AAV2-GFP-CBD was expressed. These results demonstrate that mAKAPα functions to anchor both CaN and MEF2A in the perinuclear space, and that CaN tethering is a necessary step for MEF2 binding during neuronal activity.

**Figure 8:**
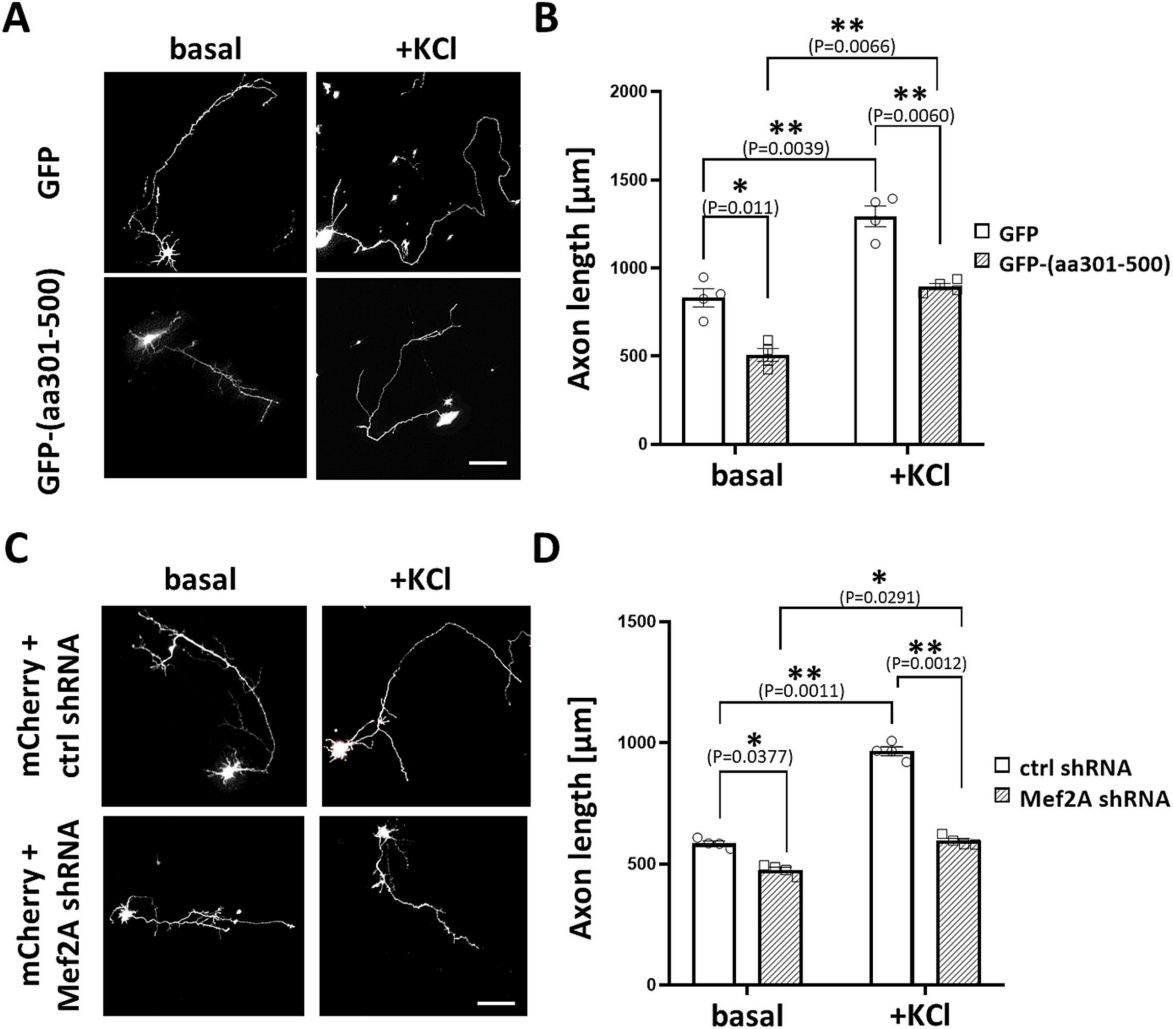
MEF2A is required for basal and KCl-induced axonal elongation. (A) Representative greyscale images for hippocampal neurons transfected with GFP control or GFP-(aa301-500) peptide. After transfection, neurons were cultured in growth media containing 5 mM KCl (basal) or 30 mM KCl (+KCl) for 24 h. (B) Quantification of axon length. Mean length of the longest neurite are shown for four (*n* = 4) independent experiments. (C) Representative grayscale micrographs of neurons co-transfected with mCherry and either MEF2A shRNA or scrambled shRNA. Neurons were cultured in the presence of 5 mM or 30 mM KCl for 24 h before assessing axonal outgrowth. (D) Analysis of changes in the average length of the longest neurite, based on *n* = 4 independent experiments. Scale bar 100 μm. *p < 0.05, **p < 0.01.

Based on these findings, we sought to investigate whether MEF2 scaffolding by mAKAPα is critical for neuronal outgrowth in hippocampal neurons. We constructed a GFP-tagged peptide that specifically displaces MEF2 from the mAKAPα complex (amino acids 301–500), as mapped elsewhere (Vargas et al., 2012). Neurons were transfected with expression plasmids for either control GFP or GFP-(aa301-500), cultured for 24 h in the presence or absence of KCl, and then assayed for neurite extension (Figures 8A,B). Cells expressing the GFP-(aa301-500) peptide exhibited significantly shorter axons under both basal and stimulated conditions. Similar phenotypic changes were observed in neurons transfected with MEF2A shRNA (Figures 8C,D). These data, along with the results from mAKAPα depletion and CaN/mAKAPα disruption studies, conclusively demonstrate that the CaN/MEF2 interaction at mAKAPα is required for activity-induced signaling with MEF2A playing a crucial role in the axonal elongation of primary hippocampal neurons.

## 4 Discussion

One of the fundamental processes during nervous system development is axonal extension in response to various stimuli. It has been demonstrated that physiological levels of electrical activity and cAMP control the trophic responsiveness of intact central nervous system (CNS) neurons, as well as their transcriptional and morphological response to injury (Shen et al., 1999). Several studies have also shown that electrical activity can enhance axon outgrowth during neuronal development and promote regeneration (Goldberg et al., 2002; Lim et al., 2016; Li et al., 2016; Varadarajan et al., 2023). Increasing evidence suggests that axonal growth and regeneration at the cellular level require sophisticated mechanisms to integrate signals within and between neuronal compartments (Winter et al., 2022; Terenzio et al., 2017). Recent findings indicate that these compartments are organized by A-kinase anchoring proteins (AKAPs), which act as molecular scaffolds for multiple signaling molecules involved in survival and death pathways (Wild and Dell'Acqua, 2018; Mackiewicz and Boczek, 2025; Tomczak et al., 2024). While the specific roles of individual AKAPs in neurons are not yet fully understood, it is hypothesized that some AKAPs serve as integration hubs for neuronal activity and neurotrophic signaling, counteracting retrograde pro-apoptotic signals and thereby supporting neuronal survival and axonal extension. We recently published that a neuronal perinuclear compartment, organized by the mAKAPα signalosome, is essential for the survival of RGCs, a type of CNS neuron, *in vivo* after injury (Boczek et al., 2021; Boczek et al., 2019). Our current study extends this model of mAKAPα compartmentalized signaling to the activity-dependent axonal outgrowth of hippocampal neurons.

Despite the well-established theory of compartmentalized signaling in neurons, it remains unclear how mAKAPα orchestrates divergent signaling pathways in the perinuclear space to regulate activity-dependent axonal elongation. In excitable cells such as cardiac myocytes, mAKAPα binds the Ca^2+^-activated protein phosphatase calcineurin (CaN) (Pare et al., 2005b; Li et al., 2010). It is well-known that CaN plays a crucial role in various neuronal processes, including neurotransmitter release, receptor function regulation, signal transduction, neurite outgrowth, gene expression, and neuronal cell death (Chen et al., 2022). Our findings reveal that, similar to myocytes, CaN interacts with mAKAPα in hippocampal neurons, and this interaction is enhanced by KCl-induced neuronal activity. However, CaN is not exclusively tethered to mAKAPα; its interaction with other scaffolds has also been explored (Li et al., 2011). For instance, CaN directly binds to AKAP79/150 in hippocampal neurons, where it regulates L-type Ca^2+^ channels and couples channel activity to NFATc4 activation (Oliveria et al., 2007). This observation suggests that the tethering of the phosphatase to different AKAPs may be physiologically relevant for controlling neuronal activity.

Our experiments with the CaN anchoring disruptor (mCherry-CBD peptide) clearly demonstrated that CaN scaffolding by mAKAPα in the perinuclear space is crucial for axonal outgrowth following KCl depolarization. Notably, the inhibition of axon growth in the presence of the anchoring disruptor was associated with increased expression of Bcl-2, a member of the anti-apoptotic protein family. This suggests that mAKAPα, by anchoring CaN, may provide localized control of CaN activity during neuronal development. It has been shown that alterations in CaN activity can prevent the morphological maturation of interneurons and inhibit apoptosis during embryonic stages (E15-P5), while increasing apoptosis in postnatal (P5-P20) neurons (Priya et al., 2018). In contrast, pharmacological inhibition of CaN promotes axonal extension in Xenopus spinal neurons in the presence of Ca^2+^ (Lautermilch and Spitzer, 2000), suggesting that the effects of manipulating CaN activity may be specific to the type of neuron and its developmental stage. Increased Bcl-2 expression, a potent inhibitor of apoptosis, is a known mechanism that supports the survival of injured neurons, as demonstrated in axotomized RGCs (Shen et al., 1999). Therefore, the increased Bcl-2 expression observed in neurons overexpressing the CaN-displacing peptide could be a protective response to counteract dysregulated CaN activity.

Our previous work demonstrated that mAKAPα specifically coordinates Ca^2+^-dependent cAMP signaling in the perinuclear space to support axonal growth *in vitro* and neuroprotection *in vivo* (Boczek et al., 2021; Boczek and Kapiloff, 2020; Boczek et al., 2019). In this study, we show that disrupting the CaN/mAKAPα interaction leads to changes in the expression of genes involved in Ca^2+^/cAMP signaling. Microarray analysis revealed that following transduction with the AAV2-GFP-CBD anchoring disruptor, the expression of five genes—*Nos2*, *Mif*, *Plat*, *Tnf*, and *Cyr61*—was significantly upregulated, while the expression of *Prkar1a* was notably downregulated. These genes play crucial roles in immune regulation, neurodevelopment, and neurotransmission. Our findings align with previous studies, such as one showing that the conditional knockout of *Prkar1a* in Schwann cells resulted in a significant and lasting defect in axonal sorting, along with an unexpected reduction in Schwann cell proliferation *in vivo* (Guo et al., 2013). Additionally, Gangoda et al. (2014) demonstrated that the loss of *Prkar1a* led to the transcriptional activation of several pro-apoptotic Bcl-2 family members, resulting in cell death. Microglia inhibitory factor (*Mif*), in turn, has been associated with demyelination and axonal degeneration in the early stages following injury (Muzio et al., 2021). Research using a mouse model of spinal cord injury showed that inhibiting microglia and macrophage activation with the tripeptide MIF/TKP reduced reactive gliosis and increased the number of axons within the lesion epicenter (Emmetsberger and Tsirka, 2012). The potential role of *Mif* in nerve regeneration was further investigated by Nishio et al. (2002) who reported that the local administration of an anti-MIF antibody into regenerating rat sciatic nerves significantly reduced nerve length compared to treatment with non-immune rabbit IgG. Another gene implicated in neuronal survival is the serine protease tPA, the primary enzyme responsible for converting plasminogen into its active form, plasmin. A growing body of evidence supports tPA’s pro-survival effects on neurons both *in vivo* and *in vitro* (Flavin & Zhao, 2001; Echeverry et al., 2010; Xin et al., 2010; Buisson et al., 1998). However, other studies have reported toxic effects of tPA or the protective effects of its endogenous inhibitor (Head et al., 2009; Flavin et al., 2000; Gabriel et al., 2003). Our study suggests that the upregulation of *Plat* in response to the CaN anchoring disruptor may be associated with reduced axonal outgrowth. Consistent with our findings, elevated *Plat* expression was observed in primary postnatal cortical neurons following activity deprivation induced by the voltage-gated sodium channel blocker TTX (Schonfeld-Dado and Segal, 2011). Furthermore, the administration of plasminogen activator inhibitor-1 (PAI-1) protected TTX-exposed neurons from cell death. Furthermore, the administration of plasminogen activator inhibitor-1 (PAI-1) protected the TTX-exposed neurons from cell death (Schonfeld-Dado and Segal, 2011). We acknowledge that the 24-h time point chosen for the microarray screening provides only a snapshot of the changes induced by the GFP-CBD anchoring disruptor. Therefore, conducting an extended analysis to capture earlier changes in the genetic profile would be valuable in uncovering the mechanisms underlying the altered axonal outgrowth observed in the presence of GFP-CBD. Collectively, our results support a model in which mAKAPα compartmentalization of CaN activity is essential for shaping cellular responses during neuronal development. While it has not yet been fully elucidated how the scaffold controls axonal outgrowth through CaN tethering, we hypothesize, based on previous findings in myocytes (Li et al., 2010), that CaN retains its activity upon binding to the scaffold, enabling it to regulate the dephosphorylation-dependent events in the perinuclear space, thereby affecting axonal growth.

To explore this hypothesis, we investigated whether any known CaN substrates bind to mAKAPα and found that NFATc4 interacts with the scaffold in a CaN- and Ca^2+^-dependent manner. Research on NFAT transcription factors suggests an important role in neuronal physiology (Schonfeld-Dado and Segal, 2011). For instance, NFATc4 activity is necessary for the survival of adult-born neurons in response to BDNF (Quadrato et al., 2012) and facilitates anti-apoptotic gene expression in cortical neurons stimulated by NMDA receptors (Vashishta et al., 2009). Supporting this, the concurrent deletion of NFATc3 and NFATc4 resulted in severe defects in sensory axon projection (Graef et al., 2001). Our recent work demonstrated that NFATc4 is specifically but transiently upregulated in response to mechanical injury, and its knockout improved the survival of injured RGCs and slowed axonal degeneration (Mackiewicz et al., 2024). Interestingly, neither NFATc4 (Mackiewicz et al., 2024) nor mAKAPα (Wang et al., 2015) was essential for the normal development of these neurons. Similarly, in primary hippocampal neurons, disrupting the NFAT/mAKAPα interaction or blocking CaN-dependent NFAT activation did not affect axonal elongation. This suggests that NFAT function is not critical for the normal development or maintenance of uninjured hippocampal neurons. Why NFATc4 is tethered to mAKAPα in response to KCl-induced neuronal activity and which neuronal processes involve mAKAPα-dependent control of NFAT transcriptional activity remain open questions.

The dynamic shuttling between the cytoplasm and nucleus allows for tight temporal and spatial control of NFAT-mediated gene transcription, playing a key role in processes such as neurite outgrowth, synaptogenesis, neuronal development, and survival (Mackiewicz et al., 2023). In general, under resting conditions, NFAT proteins are located in the cytoplasm and remain in inactive state (Vaeth and Feske, 2018). Upon an increase in intracellular Ca^2+^ levels, CaN becomes activated and dephosphorylates NFAT, promoting its rapid translocation into the nucleus. Within the nuclear environment, NFAT serves as a mediator that connects intracellular Ca^2+^ dynamics to the regulation of gene transcription, functioning either independently or, more frequently, in conjunction with other transcription factors (Flanagan et al., 1991). The dephosphorylation of NFAT is counteracted by the activity of various kinases that either maintain NFAT in a phosphorylated state in the cytosol to prevent its nuclear import or re-phosphorylate NFAT within the nucleus, promoting its nuclear export (Mackiewicz et al., 2023). In our study, we propose a new molecular mechanism by which the association of CaN with mAKAPα regulates NFATc4 transcriptional activity by promoting the dephosphorylated state of NFATc4 and its nuclear localization, thereby facilitating its cooperation with other transcription factors during neuronal development. A similar observation was reported by Li et al. (2010), who demonstrated that blocking CaN tethering to mAKAPβ in rat neonatal cardiomyocytes attenuated the dephosphorylation of NFATc3 transcription factors, suggesting a more generalized mechanism regulating NFAT activity. It is, therefore, tempting to speculate that mAKAP located in the perinuclear space may provide additional control over NFATc4 activity during its nuclear import. This localized regulation of NFATc4—and potentially other NFAT family members—could enable compartment-specific phosphorylation and dephosphorylation of NFAT. Its interaction with the mAKAP signalosome machinery could act as a critical checkpoint for NFAT activation just before its entry into the nucleus.

Another key effector of CaN in neurons is the transcription factor myocyte enhancer factor 2 (MEF2). Our studies revealed that neuronal activity is linked to MEF2A scaffolding in the perinuclear compartment of primary rat hippocampal neurons. The significance of this interaction for axonal outgrowth is underscored by the finding that disrupting MEF2 anchoring to mAKAPα led to decreased neuronal outgrowth under both basal and KCl-stimulated conditions. Research on primary cerebellar granule neurons has demonstrated the pro-survival role of MEF2, showing that MEF2A hairpin RNAs suppress activity-dependent granule neuron survival (Gaudilliere et al., 2002). Similarly, Li et al. (2001) found that expressing a constitutively active form of MEF2 promoted the survival of cultured cerebellar granule neurons during apoptosis. Further supporting the pro-survival function of MEF2, *in vivo* studies have shown that brain-specific deletion of MEF2A/C/D in mice leads to early postnatal death, accompanied by increased neuronal apoptosis (Akhtar et al., 2012). Conversely, MEF2—particularly MEF2A and MEF2C—may play a detrimental role in the survival of injured axons. Recently, Xia et al. (2020) reported a significant increase in mRNA levels for Mef2a and Mef2c five days after optic nerve crush. Using a loss-of-function approach, they demonstrated that targeting Mef2a is sufficient to confer neuroprotection to retinal ganglion cells (RGCs). These findings suggest that MEF2 transcription factors may act as a switch between axonal growth and degeneration, depending on the developmental stage and neuronal integrity. An open question remains regarding the mechanisms that regulate the switch between the dual roles of MEF2. Our study suggests that neuronal mAKAPα may provide an effective platform for the enzymes involved in regulating MEF2 activation. Direct binding of MEF2, along with its interaction with specific signaling pathways and co-regulatory proteins anchored to the perinuclear space by the mAKAP signalosome, could offer precise spatial and temporal control over MEF2’s active and inactive states. This local regulation might be achieved through mechanisms such as CaN-dependent MEF2 dephosphorylation. For instance, studies on hippocampal neurons have shown that CaN promotes the dephosphorylation of MEF2A, thereby enhancing its transactivation activity, which is essential for calcium-dependent neuronal dendritic outgrowth (Shalizi et al., 2006). Additionally, the dynamic regulation of MEF2 localization is influenced by its interaction with proteins such as histone deacetylases (HDACs). Specifically, class II HDACs (HDACs 4, 5, 7, and 9) bind to MEF2 in the nucleus, repressing its activity by inhibiting access to its target gene (McKinsey et al., 2002). In contrast to the NFATc4-mAKAPα interaction, MEF2A binding to the signalosome in the perinuclear space is part of a signaling pathway through which neuronal activity influences axonal outgrowth during development. While the perinuclear regulation of MEF2 activity appears to play a critical role in this process, further research is needed to elucidate how mAKAPα controls MEF2-dependent gene transcription in neurons. These findings underscore the role of mAKAPα as a crucial nodal point in neuronal signaling, serving as a key regulator not only of NFAT but also of MEF2 transcription factors.

In conclusion, the perinuclear space organized by mAKAPα signalosome is an important neuronal compartment for activity-dependent outgrowth of hippocampal neurons. Taking the results of disruption and shRNA studies under consideration, we can draw a model where mAKAPα by bringing together CaN and MEF2 in the perinuclear space provides a local control for transcription factor activity to promote the expression of genes involved in axonal elongation.

## Data Availability

The raw data supporting the conclusions of this article will be made available by the authors, without undue reservation. Microarray data presented in the study are deposited in the ArrayExpress repository, accession number E-MTAB-14614.
