# Supplementary material for: Perinuclear compartment controls calcineurin/MEF2 signaling for axonal outgrowth of hippocampal neurons
Source: Front Mol Neurosci. 2024 Nov 25;17:1494160. doi: 10.3389/fnmol.2024.1494160 (PMC11625814; doi:10.3389/fnmol.2024.1494160)
Supplement: Supplementary file 1 [file Data_Sheet_1.pdf]

Supplementary Materials

1. Supplementary Tables

Supplementary Tab.1. The list of genes screened for cAMP/Ca^{2+} signaling. The experiment was performed in triplicate and the data were analyzed using SABiosciences PCR Array Data Analysis Web Portal as described in Materials and Methods section.

GENE BANK	GENE SYMBOL	DESCRIPTION
**NM_012701**	Adrb1	Adrenergic, beta-1-, receptor
**NM_013149**	Ahr	Aryl hydrocarbon receptor
**NM_031011**	Amd1	Adenosylmethionine decarboxylase 1
**NM_017123**	Areg	Amphiregulin
**NM_012912**	Atf3	Activating transcription factor 3
**NM_016993**	Bcl2	B-cell CLL/lymphoma 2
**NM_012513**	Bdnf	Brain-derived neurotrophic factor
**NM_012514**	Brca1	Breast cancer 1
**NM_031984**	Calb1	Calbindin 1
**NM_053988**	Calb2	Calbindin 2
**NM_012717**	Calcrl	Calcitonin receptor-like

**NM_031969**	Calm1	Calmodulin 1
**NM_022399**	Calr	Calreticulin
**NM_001011949**	Ccna1	Cyclin A1
**NM_171992**	Ccnd1	Cyclin D1
**NM_080885**	Cdk5	Cyclin-dependent kinase 5
**NM_130812**	Cdkn2b	Cyclin-dependent kinase inhibitor 2B (p15, inhibits CDK4)
**NM_053918**	Cga	Glycoprotein hormones, alpha polypeptide
**NM_021655**	Chga	Chromogranin A
**NM_031747**	Cnn1	Calponin 1, basic, smooth muscle
**NM_031017**	Creb1	CAMP responsive element binding protein 1
**NM_001110860**	Crem	CAMP responsive element modulator
**NM_031019**	Crh	Corticotropin releasing hormone
**NM_017129**	Ctf1	Cardiotrophin 1
**NM_031327**	Cyr61	Cysteine-rich, angiogenic inducer, 61
**NM_024134**	Ddit3	DNA-damage inducible transcript 3
**NM_053769**	Dusp1	Dual specificity phosphatase 1

**NM_012551**	Egr1	Early growth response 1
**NM_053633**	Egr2	Early growth response 2
**NM_139325**	Eno2	Enolase 2, gamma, neuronal
**NM_131908**	Fgf6	Fibroblast growth factor 6
**NM_022197**	Fos	FBJ osteosarcoma oncogene
**NM_012707**	Gcg	Glucagon
**NM_001106637**	Gem	GTP binding protein (gene overexpressed in skeletal muscle)
**NM_012714**	Gipr	Gastric inhibitory polypeptide receptor
**NM_012735**	Hk2	Hexokinase 2
**NM_153629**	Hspa4	Heat shock protein 4
**NM_053836**	Il2	Interleukin 2
**NM_012589**	Il6	Interleukin 6
**NM_017128**	Inhba	Inhibin beta-A
**NM_021836**	Junb	Jun B proto-oncogene
**NM_138875**	Jund	Jun D proto-oncogene
**NM_012972**	Kcna5	Potassium voltage-gated channel, shaker-related subfamily, member 5

**NM_017025**	Ldha	Lactate dehydrogenase A
**NM_019318**	Maf	V-maf musculoaponeurotic fibrosarcoma oncogene homolog (avian)
**NM_031051**	Mif	Macrophage migration inhibitory factor
**NM_031521**	Ncam1	Neural cell adhesion molecule 1
**NM_012609**	Nf1	Neurofibromin 1
**NM_012611**	Nos2	Nitric oxide synthase 2, inducible
**NM_012614**	Npy	Neuropeptide Y
**NM_019328**	Nr4a2	Nuclear receptor subfamily 4, group A, member 2
**NM_001108377**	Pck2	Phosphoenolpyruvate carboxykinase 2 (mitochondrial)
**NM_022381**	Pcna	Proliferating cell nuclear antigen
**NM_017139**	Penk	Proenkephalin
**NM_001034125**	Per1	Period homolog 1 (Drosophila)
**NM_013151**	Plat	Plasminogen activator, tissue
**NM_022707**	Pln	Phospholamban
**NM_001008385**	Pmaip1	Phorbol-12-myristate-13-acetate-induced protein 1
**NM_013008**	Pou1f1	POU class 1 homeobox 1

**NM_001109599**	Pou2af1	POU class 2 associating factor 1
**NM_133546**	Ppp1r15a	Protein phosphatase 1, regulatory (inhibitor) subunit 15A
**NM_017039**	Ppp2ca	Protein phosphatase 2, catalytic subunit, alpha isoform
**NM_013181**	Prkar1a	Protein kinase, cAMP-dependent, regulatory, type I, alpha
**NM_012629**	Prl	Prolactin
**NM_017232**	Ptgs2	Prostaglandin-endoperoxide synthase 2
**NM_017045**	Rb1	Retinoblastoma 1
**NM_053485**	S100a6	S100 calcium binding protein A6
**NM_053587**	S100a9	S100 calcium binding protein A9
**NM_012521**	S100g	S100 calcium binding protein G
**NM_022669**	Scg2	Secretogranin II (chromogranin C)
**NM_019232**	Sgk1	Serum/glucocorticoid regulated kinase 1
**NM_013152**	Slc18a1	Solute carrier family 18 (vesicular monoamine), member 1
**NM_017051**	Sod2	Superoxide dismutase 2, mitochondrial
**NM_001109302**	Srf	Serum response factor (c-fos serum response element-binding transcription factor)
**NM_012659**	Sst	Somatostatin

**NM_019348**	Sstr2	Somatostatin receptor 2
**NM_012747**	Stat3	Signal transducer and activator of transcription 3
**NM_012667**	Tacr1	Tachykinin receptor 1
**NM_013174**	Tgfb3	Transforming growth factor, beta 3
**NM_012740**	Th	Tyrosine hydroxylase
**NM_001013062**	Thbs1	Thrombospondin 1
**NM_012675**	Tnf	Tumor necrosis factor (TNF superfamily, member 2)
**NM_001107248**	Vcl	Vinculin
**NM_053991**	Vip	Vasoactive intestinal peptide
**NM_031144**	Actb	Actin, beta
**NM_012512**	B2m	Beta-2 microglobulin
**NM_012583**	Hprt1	Hypoxanthine phosphoribosyltransferase 1
**NM_017025**	Ldha	Lactate dehydrogenase A
**NM_001007604**	Rplp1	Ribosomal protein, large, P1

2. Supplementary Figures

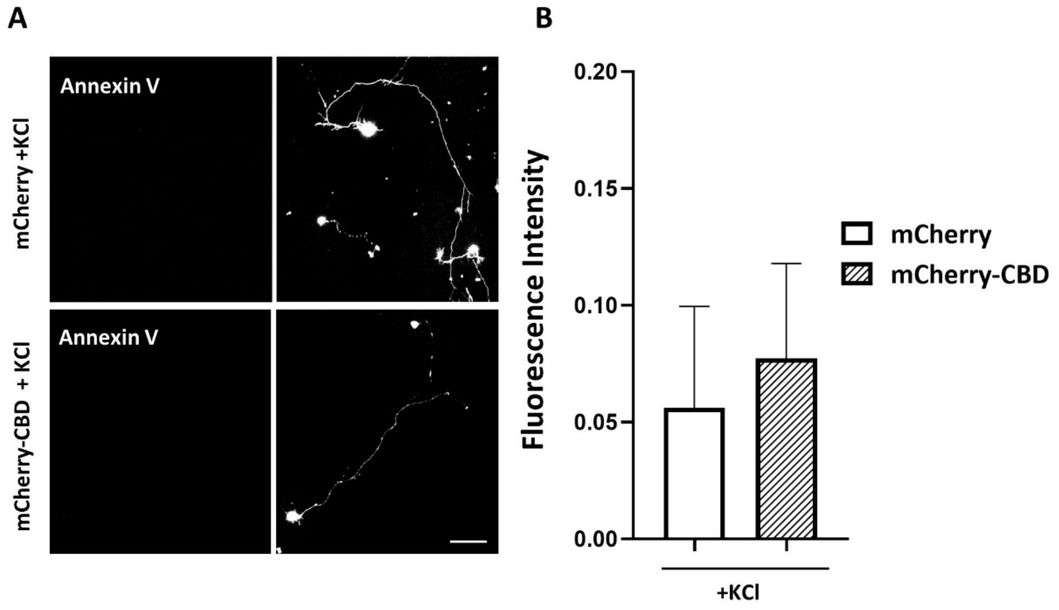

Supplementary Fig. 1. Representative grayscale images of neurons expressing mCherry or mCherry-CBD stained for Annexin V to visualize apoptotic cells. Annexin V fluorescence was assayed 24 hours following stimulation with 30 mM KCl. Scale bar: 100 µm. (B) Quantification of Annexin V-positive neurons. The average fluorescence from n=8 cells was measured in triplicate using LAS X software for Leica microscopes.

Supplementary Figure 1. Representative grayscale images of neurons expressing mCherry or mCherry-CBD stained for Annexin V to visualize apoptotic cells. Annexin V fluorescence was assayed 24 hours following stimulation with 30 mM KCl. Scale bar: 100 µm. (B) Quantification of Annexin V-positive neurons. The average fluorescence from n=8 cells was measured in triplicate using LAS X software for Leica microscopes.

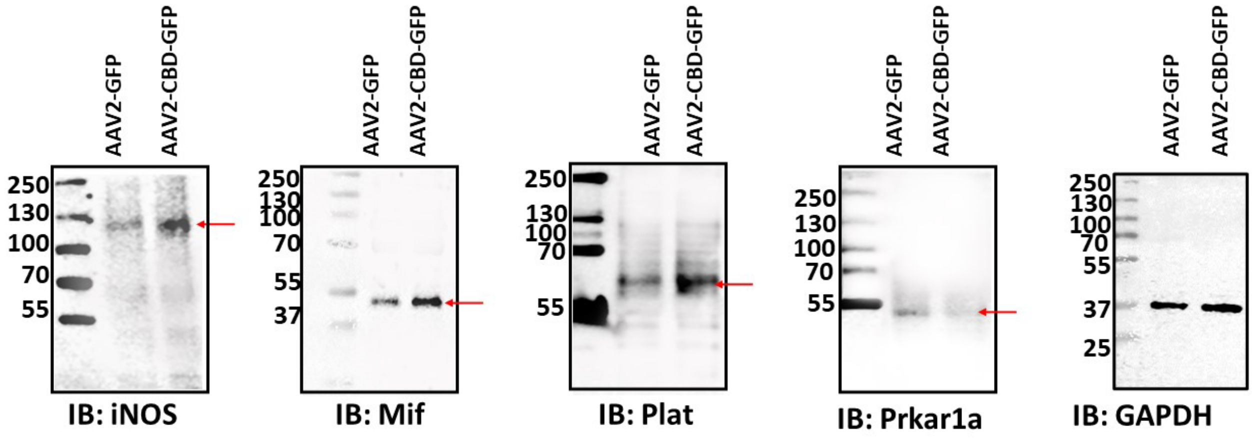

Supplementary Fig. 2. Representative full-size immunoblots demonstrating changes in protein levels following transduction with AAV2-CBD-GFP or AAV2-GFP control. The arrow indicates the band chosen for densitometric scanning. The quantification is presented in Fig. 4.

Supplementary Figure 2. Representative full-size immunoblots demonstrating changes in protein levels following transduction with AAV2-GFP-CBD or AAV2-GFP control. The arrow indicates the band chosen for densitometric scanning. The quantification is presented in Fig. 4.
